# Integrative Transcriptomic Analysis Identifies a Key Candidate Gene Bridging Inflammation and DNA Repair in Endometriosis

**DOI:** 10.1155/sci/7134066

**Published:** 2026-07-21

**Authors:** Juan Wang, Wangshu Li, Jiuxiang Feng, Zhongmin Wang

**Affiliations:** ^1^ Department of Gynecology, Dalian Medical University, Dalian 116021, Liaoning Province, China, dlmedu.edu.cn; ^2^ Department of Gynecology, Dalian Women and Children’s Medical Group, Dalian 116012, Liaoning Province, China

**Keywords:** DNA repair, endometriosis, immune infiltration, inflammation, transcriptomics

## Abstract

**Background:**

Endometriosis is a chronic, estrogen‐dependent inflammatory disorder characterized by the ectopic implantation of endometrial‐like tissue. Although retrograde menstruation is highly prevalent, only a subset of women develops the disease. This epidemiologic paradox suggests that intrinsic molecular alterations in the eutopic endometrium may precondition refluxed cells to survive under inflammatory and oxidative stress.

**Methods:**

Eutopic endometrial transcriptomes from the GSE6364 dataset (21 endometriosis patients and 16 controls) were analyzed across menstrual phases. Differential expression analysis, Gene Ontology (GO) and Kyoto Encyclopedia of Genes and Genomes (KEGG) enrichment, and hallmark inflammatory gene set profiling were performed. Protein–protein interaction (PPI) networks and three machine‐learning algorithms maximal clique centrality (MCC), random forest (RF), and least absolute shrinkage and selection operator (LASSO) were applied to identify robust feature genes. Immune cell infiltration was estimated using CIBERSORT . Excision repair cross‐complementation group 1 (ERCC1) was further evaluated via cross‐species evolutionary conservation and in silico structural modeling of high‐risk variants. Additionally, an exploratory stemness–related single‐sample gene set enrichment analysis (ssGSEA) was conducted, and correlations between the stemness score and the feature genes were examined.

**Results:**

A total of 443 differentially expressed genes (DEGs) were identified, which were significantly enriched in inflammatory cascades, including the NF‐κB, Toll‐like receptor, and cytokine signaling pathways. Four convergent feature genes ERCC1, SOX3, P75NTR (encoded by NGFR), and FPR1 were prioritized. Immune deconvolution revealed selective immune remodeling in the eutopic endometrium, characterized by elevated activated natural killer (NK) cells and reduced CD8^+^ T cells, which correlated significantly with the expression of the feature genes. ERCC1 exhibited high evolutionary conservation, and structural modeling of missense variants predicted the disruption of protein function within conserved DNA repair domains. Exploratory stemness analysis revealed no significant overall case–control difference after adjusting for menstrual phase, though the early secretory subset exhibited a marginally higher score in endometriosis.

**Conclusions:**

These findings highlight a stress‐adaptive transcriptomic state in the eutopic endometrium driven by inflammatory signaling, selective immune remodeling, and altered DNA repair capacity. Specifically, ERCC1 may serve as a critical mechanistic link between inflammatory pressure and impaired genomic maintenance, thereby facilitating cellular persistence and lesion establishment. Furthermore, our data indicate that this four‐gene signature primarily reflects a DNA‐repair‐adaptive program rather than a global bulk‐tissue stemness shift.

## 1. Introduction

Endometriosis is a chronic, estrogen‐dependent inflammatory disorder defined by the ectopic presence of endometrial glands and stroma, most commonly on the pelvic peritoneum and ovaries. It is tightly linked to pelvic pain and infertility. Affecting 6%–10% of reproductive‐age women, it imposes a substantial diagnostic delay and long‐term morbidity, reflecting both symptom heterogeneity and a continued reliance on invasive diagnostic approaches [1, 2].

The most widely cited etiologic framework, Sampson’s retrograde menstruation theory, provides a compelling physical route for endometrial tissue displacement. However, while retrograde menstruation is nearly universal in women with patent fallopian tubes, only a minority develop endometriosis—an epidemiologic paradox implying the existence of additional permissive factors beyond tissue reflux [3, 4]. This discrepancy has renewed focus on the “seed‐and‐soil” concept: the eutopic endometrium of affected individuals may be intrinsically primed for survival, adhesion, invasion, and immune evasion, thereby converting a common physiological event into a pathological implantation cascade [5].

The peritoneal microenvironment in endometriosis is defined by a persistent “cytokine storm” and chronic oxidative stress, driven by periodic hemorrhage, iron overload, and macrophage activation [6]. This inflammatory milieu imposes relentless genotoxic stress on retrograde endometrial cells, generating reactive oxygen species (ROS) that constantly threaten genomic integrity [7]. Under normal physiological conditions, such accumulated DNA damage triggers cell cycle arrest or apoptosis. However, endometriotic cells develop mechanisms to evade this surveillance, suggesting an aberrant adaptation in their DNA damage response (DDR) machinery that favors cell survival over genomic fidelity [8].

Nucleotide excision repair (NER) is a primary defense mechanism against bulky DNA adducts and oxidative lesions. Within this pathway, the excision repair cross‐complementation group 1 (ERCC1) acts as a rate‐limiting endonuclease essential for maintaining genomic stability [9]. Crucially, recent genomic landscape studies have fundamentally shifted our understanding of the disease, revealing that even benign endometriotic lesions and eutopic endometrium harbor recurrent somatic mutations [5, 10]. These observations suggest that compromised DNA repair, whether driven by transcriptional dysregulation or intrinsic structural variations, may be integral to lesion biology. This preexisting vulnerability likely drives persistence and repeated lesion outgrowth, which is consistent with epidemiologic evidence linking endometriosis to an elevated risk of certain malignancies, such as ovarian cancer [11, 12].

Despite these insights, the precise molecular crosstalk between inflammatory signaling and DNA repair capacity remains poorly understood. To bridge this gap, we applied an integrative computational strategy. First, we utilized eutopic endometrial transcriptomics and machine‐learning feature selection to identify core genes linking inflammatory stress to genome maintenance. Next, recognizing that functional impairment of DNA repair enzymes can arise from genetic polymorphisms, we employed in silico structural modeling to evaluate how high‐risk genetic variants physically compromise the identified target proteins. We propose that the synergistic effect of inflammation‐associated transcriptional dysregulation and structural instability compromises genomic integrity, thereby facilitating the establishment and persistence of endometriosis.

## 2. Materials and Methods

### 2.1. Data Acquisition and Transcriptomic Profiling

To investigate the molecular landscape of endometriosis, gene expression profiles were obtained from the Gene Expression Omnibus (GEO) database. The dataset GSE6364 served as the primary cohort, comprising eutopic endometrial biopsy samples from women with laparoscopy‐confirmed moderate‐to‐severe endometriosis (*n* = 21) and control women without endometriosis (*n* = 16), collected across the proliferative, early secretory, and mid‐secretory phases of the menstrual cycle [13]. Raw data were preprocessed and normalized using the affy R package.

For differential expression analysis, the endometriosis samples were compared directly with the nonendometriosis eutopic endometrial samples within the dataset. This case–control design was intentionally selected to characterize the transcriptomic state of the eutopic endometrium under inflammatory stress—which may predispose cells to survival and ectopic implantation—rather than to define lesion‐specific ovarian expression patterns. Differential expression analysis was performed using the limma package, with a statistical significance threshold set at an adjusted *p*‐value <0.05 and an absolute log2 fold change (|log2FC|) > 0.5.

#### 2.1.1. Functional Enrichment and Stemness‐Related Scoring

To decipher the biological context of the identified differentially expressed genes (DEGs), gene set enrichment analysis (GSEA) and gene set variation analysis (GSVA) were conducted to evaluate inflammatory response signatures. Functional annotation was performed using the ClusterProfiler package to identify enriched Gene Ontology (GO) terms and Kyoto Encyclopedia of Genes and Genomes (KEGG) pathways.

To further assess whether the observed transcriptomic alterations were accompanied by progenitor‐like properties, we performed an exploratory single‐sample GSEA (ssGSEA) using gene sets annotated to stem cell maintenance (GO:0019827) and somatic stem cell population maintenance (GO:0035019). The resulting stemness‐related scores were compared between case and control samples across the overall cohort and within menstrual‐phase strata. A phase‐adjusted linear model was fitted to account for cycle‐dependent effects, followed by Spearman correlation analyses between the stemness score and candidate feature genes.

### 2.2. Machine‐Learning Feature Selection and Immune Deconvolution

A multialgorithm machine‐learning pipeline was implemented to prioritize robust candidate feature genes linking inflammation to cellular stress responses. First, a protein–protein interaction (PPI) network of DEGs was constructed using STRING (version 12), and the cytoHubba plugin in Cytoscape was used to identify hub genes based on maximal clique centrality (MCC). In parallel, a random forest (RF) model ranked genes by variable importance, and least absolute shrinkage and selection operator (LASSO) regression (with 10‐fold cross‐validation) was applied for feature penalization and selection. Genes consistently highlighted across all three algorithms (MCC, RF, and LASSO) were defined as core feature genes. Additionally, immune infiltration in the tissue microenvironment was estimated using the CIBERSORT algorithm to quantify 22 distinct immune cell subsets, and their relative abundances were correlated with the core feature genes.

### 2.3. In Silico Structural Modeling and Variant Analysis of ERCC1

Given that the GSE6364 dataset provides transcriptomic profiles without paired germline genotyping, we adopted an in silico approach to investigate potential structural vulnerabilities of the prioritized target, ERCC1. Independent of the transcriptomic cohort, high‐risk coding‐region single nucleotide polymorphisms (SNPs) of the human ERCC1 gene were retrieved from the NCBI public repository and pertinent literature [14].

To visualize the structural consequences of these variants, the crystal structure of the human ERCC1 protein was retrieved from the Protein Data Bank (PDB). Homology modeling of both wild‐type and mutant proteins was performed using Phyre2, SWISS‐MODEL, and the I‐TASSER server, utilizing hierarchical threading to identify optimal templates. Generated models were refined using the conjugate gradient approach and the Amber force field within UCSF Chimera to minimize energy and stabilize conformation.

To map functional disruptions, ligand‐binding residues were predicted using the COACH meta‐server (integrating TM‐SITE and S‐SITE based on the BioLiP database) and the FTSite server. Secondary structures were predicted using the PSIPRED server (version 3.3). Finally, to assess communication networks within the DNA repair machinery, targeted PPI analyses were visualized using Cytoscape, and biochemical conservation from primary to quaternary levels was evaluated using the ENDscript 2 server.

### 2.4. Statistical Analysis and Software

All statistical and bioinformatics analyses were conducted using R software (version 4.2.2). Key R packages utilized included affy (v1.76.0), limma (v3.54.0), ClusterProfiler (v4.6.0), randomForest (v4.7‐1.1), and glmnet (v4.1‐6). Structural analyses employed Cytoscape (v3.9.1) and UCSF Chimera (v1.16).

## 3. Results

### 3.1. Transcriptomic Profiling and Functional Enrichment Analysis of Endometriosis

Differential expression analysis was performed to identify global gene expression alterations in endometriosis. A total of 443 DEGs were identified between the endometriosis and control groups, comprising 121 upregulated and 322 downregulated genes, as illustrated in the volcano plot (Figure 1A). GSEA indicated a significant enrichment of the inflammatory response gene set in the endometriosis group (Figure 1B), confirming a heightened proinflammatory transcriptomic state. Intersection analysis between the 443 DEGs and a predefined set of immune‐related response genes (IRRGs) yielded 20 overlapping core genes (Figure 1D). Functional enrichment analysis of these intersection genes using GO and KEGG databases revealed significant associations with classical inflammatory cascades. The bubble plot and heatmap display pronounced enrichment in biological processes such as “response to lipopolysaccharide” and “regulation of inflammatory response,” alongside key signaling pathways including the NF‐κB, Toll‐like receptor, and cytokine–cytokine receptor interaction pathways (Figure 1C,E).

Figure 1Transcriptomic profile and inflammatory landscape of endometriosis. (A) Volcano plot illustrating DEGs between the endometriosis and control groups; red dots represent upregulated genes, and cyan dots represent downregulated genes. (B) GSEA showing significant enrichment of the “HALLMARK_INFLAMMATORY_RESPONSE” gene set in the endometriosis group relative to the control group, indicating a proinflammatory transcriptomic signature in endometriosis. (C) Bubble plots and heatmaps of GO and KEGG enrichment analyses revealing that DEGs are significantly enriched in inflammation‐related pathways, including response to lipopolysaccharide, NF‐κB signaling pathway, and cytokine–cytokine receptor interaction. (D) Venn diagram displaying the intersection of 443 DEGs with IRRGs, identifying 20 core inflammatory genes. (E) Heatmap illustrating the expression profiles of the 20 overlapping inflammatory genes across endometriosis and control samples.

**Figure:**
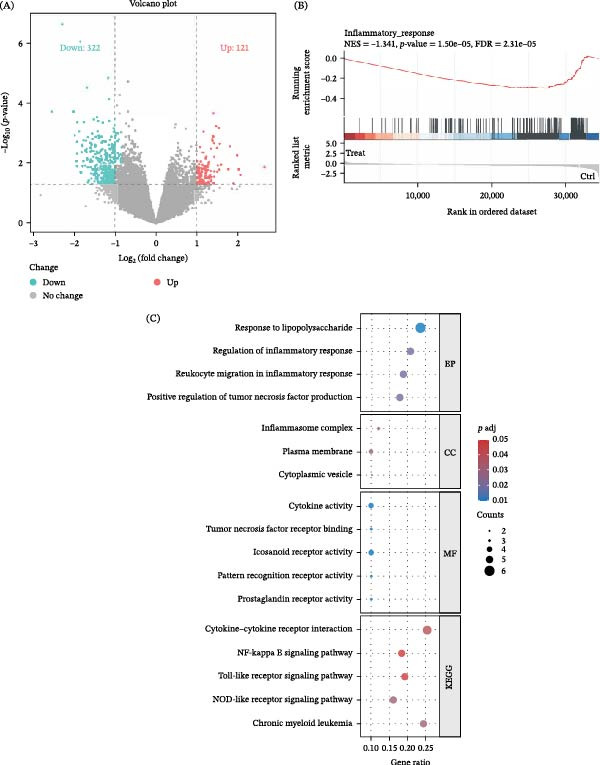

**Figure:**
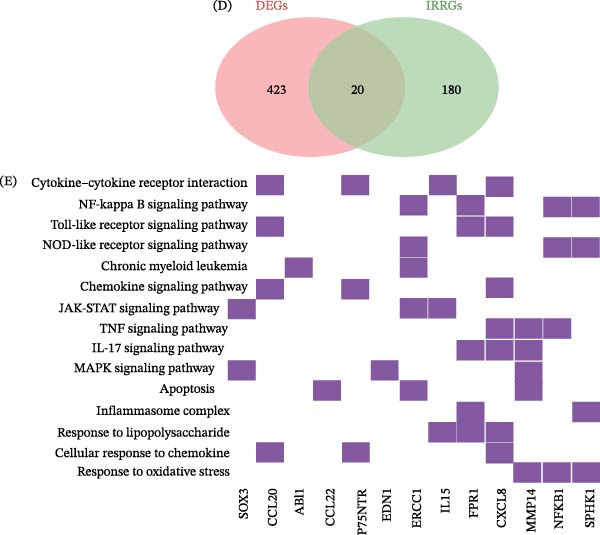

### 3.2. Screening of Candidate Feature Genes Using Machine Learning Algorithms

**Figure 2:**
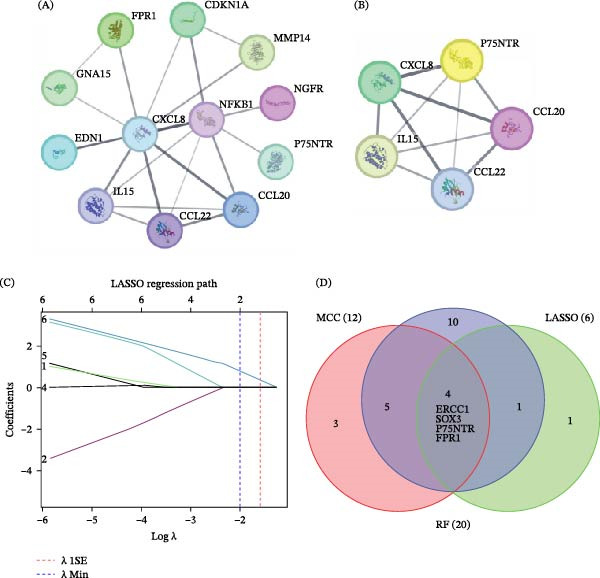
Screening of candidate feature genes via machine learning algorithms. (A) Protein–protein interaction (PPI) network of DEGs constructed based on the STRING database. (B) Top 5 key hub genes identified using the MCC algorithm within the CytoHubba plugin. (C) Feature variable selection via LASSO regression analysis, displaying the relationship between cross‐validation error and Lambda values. (D) Venn diagram showing the intersection of screening results from MCC, random forest (RF), and LASSO algorithms.

PPI analysis indicated that the overlapping candidate genes formed a coordinated inflammatory network rather than acting as isolated factors (Figure 2A). Within this network, CXCL8 and NFKB1 occupied central positions and showed strong connectivity with IL15, CCL20, CCL22, and P75NTR, suggesting that these nodes function as primary regulators of inflammatory amplification and immune‐cell recruitment. Further module extraction identified a densely connected subnetwork comprising CXCL8, IL15, P75NTR, CCL20, and CCL22 (Figure 2B), supporting the presence of a core immune‐inflammatory module associated with endometriosis.

To reduce dimensionality and isolate the most informative predictors, we applied LASSO regression. As the penalization parameter increased, a stable subset of features was retained near the optimal lambda, ultimately selecting six candidate feature genes (Figure 2C). We then integrated this LASSO‐derived set with features identified by RF analysis (20 genes) and the MCC algorithm (12 genes). The intersection of these three distinct machine‐learning approaches yielded four robust key genes: ERCC1, SOX3, P75NTR (encoded by NGFR), and FPR1 (Figure 2D).

### 3.3. Immune Cell Infiltration and Correlation With Feature Genes

Comparative analyses revealed selective alterations in immune composition within the eutopic endometrium. Specifically, the endometriosis group exhibited a higher proportion of activated natural killer (NK) cells and a concurrent reduction in CD8^+^ T cells, accompanied by a significant shift in memory B cells; most other immune subsets remained comparable between groups (Figure 3A). Unsupervised hierarchical clustering highlighted marked interindividual heterogeneity, with only partial separation between the clinical groups, largely driven by these highly variable immune subsets (Figure 3B). Furthermore, correlation analysis demonstrated coordinated relationships among specific immune cell fractions, indicative of organized immune remodeling programs rather than independent fluctuations of isolated cell types (Figure 3C,D).

Figure 3Immune cell infiltration characteristics and correlation with feature genes. (A) Boxplot displaying the abundance differences of 22 immune cell types between endometriosis and control groups analyzed using the CIBERSORT algorithm; asterisks indicate statistical significance (*p*  < 0.05 and *p*  < 0.01). (B) Dot plot representing the mean abundance of immune cell infiltration. (C) Heatmap showing the correlations among different immune cell types. (D) Box plots showing the expression levels of the four feature genes in endometriosis versus control samples. (E) Correlation heatmap analysis between the four feature genes and infiltrating immune cells, where color intensity represents the correlation coefficient.

**Figure:**
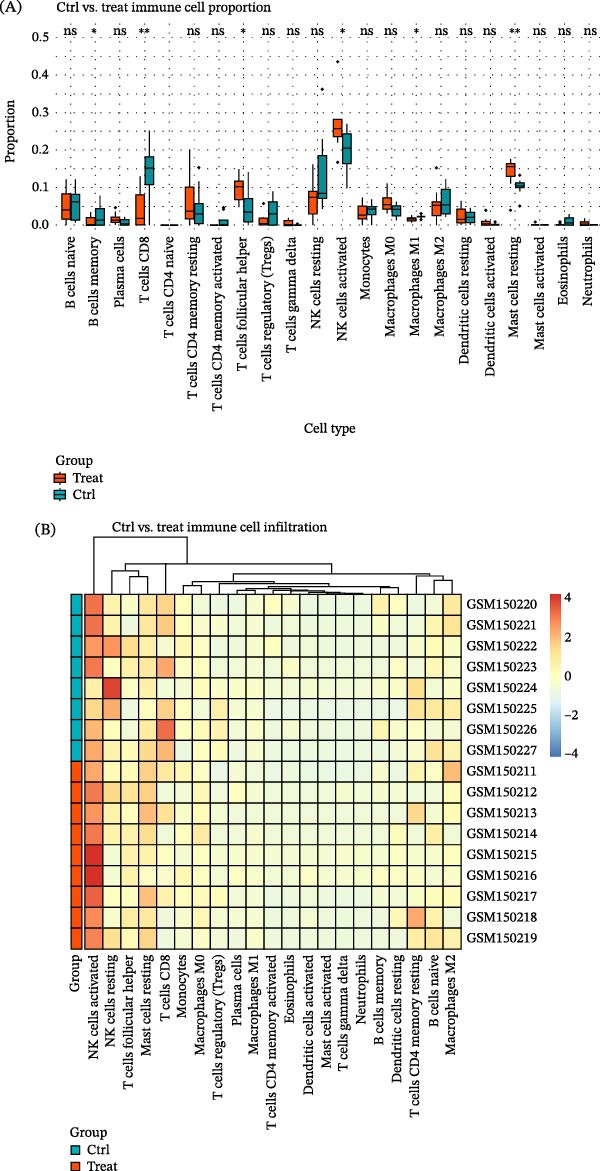

**Figure:**
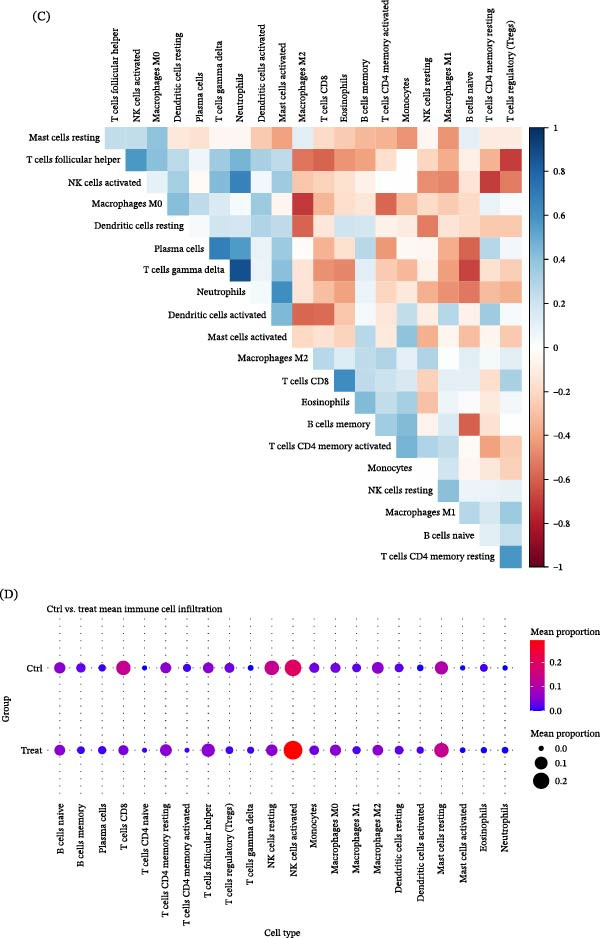

**Figure:**
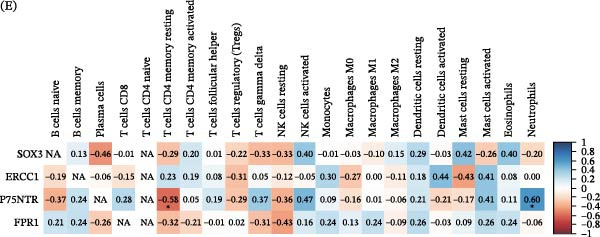

Evaluating the four prioritized feature genes revealed distinct immune‐association patterns (Figure 3E). SOX3 showed the strongest inverse correlation with plasma cells (*r* = −0.46) and a positive correlation with resting mast cells (*r* = 0.42). ERCC1 was positively correlated with activated dendritic cells (*r* = 0.44) and negatively correlated with resting mast cells (*r* = −0.43). P75NTR displayed the most pronounced immune interactions, marked by a robust negative correlation with resting CD4^+^ memory T cells (*r* = −0.58) and a strong positive correlation with neutrophils (*r* = 0.60). FPR1 was inversely associated with resting NK cells (*r* = −0.43) and positively correlated with resting dendritic cells (*r* = 0.26). Collectively, these findings suggest that the defined transcriptomic signature is intimately linked to specific immune cell compartmentalization in endometriosis.

### 3.4. Exploratory Stemness–Related Scoring Analysis

**Figure 4:**
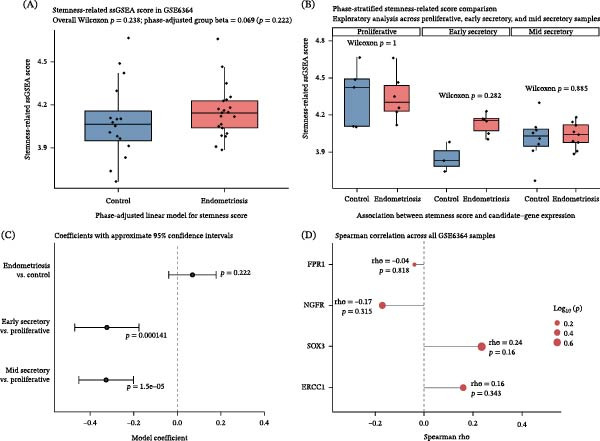
Exploratory stemness–related ssGSEA analysis in GSE6364. (A) Overall stemness‐related ssGSEA score comparison between control and endometriosis samples. (B) Phase‐stratified comparison across proliferative, early secretory, and mid‐secretory samples. (C) Phase‐adjusted linear model showing estimated group and menstrual‐phase coefficients with approximate 95% confidence intervals. (D) Spearman correlation analysis between the stemness‐related ssGSEA score and the expression of candidate genes (ERCC1, SOX3, NGFR, and FPR1) across all samples.

To further contextualize the proposed cell‐survival framework, we performed an exploratory stemness–related ssGSEA analysis in the GSE6364 cohort. At the overall cohort level, the stemness‐related score did not differ significantly between endometriosis and control samples (Wilcoxon *p* = 0.238), and this lack of a group effect persisted after adjusting for the phase of the menstrual cycle (beta = 0.069, *p* = 0.222; Figure 4A,C). Phase‐stratified analysis revealed no detectable difference in the proliferative (*p* = 1.000) or mid‐secretory (*p* = 0.885) samples, though the early secretory subset exhibited a marginally higher stemness score in the endometriosis group (*p* = 0.0282; Figure 4B).

Critically, correlation analysis demonstrated that the expression levels of the four prioritized candidate genes were not significantly associated with the overall stemness‐related score (ERCC1: rho = 0.16, *p* = 0.343; SOX3: rho = 0.24, *p* = 0.160; NGFR: rho = −0.17, *p* = 0.315; FPR1: rho = −0.04, *p* = 0.818; Figure 4D). Taken together, these results indicate that at the bulk‐transcriptome level, the identified gene signature reflects an adaptation strictly related to inflammation and DNA repair, rather than an underlying uniform enrichment of stem‐like transcriptional properties.

### 3.5. Association Between Core Feature Genes and Stemness Signatures

**Figure 5:**
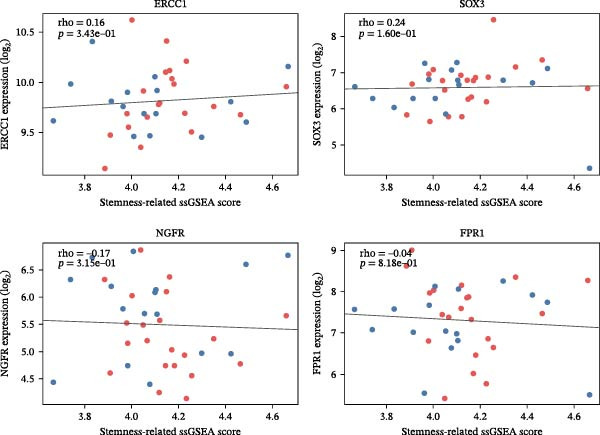
Association between stemness‐related ssGSEA score and candidate‐gene expression in GSE6364. Scatter plots illustrating the Spearman correlations between the stemness‐related ssGSEA score and the transcript levels of ERCC1, SOX3, NGFR, and FPR1. Each data point represents an individual eutopic endometrial sample.

Furthermore, to determine if the identified inflammatory and DNA repair signature is inherently coupled to progenitor‐like cellular states, we conducted correlation analyses between the overall stemness‐related ssGSEA score and the expression levels of the four prioritized candidate genes. The results demonstrated no significant correlations across the cohort (ERCC1: rho = 0.16, *p* = 0.343; SOX3: rho = 0.24, *p* = 0.160; NGFR: rho = −0.17, *p* = 0.315; FPR1: rho = −0.04, *p* = 0.818; Figure 5). Collectively, these findings reinforce the conclusion that at the bulk‐transcriptome level, the defined four‐gene signature strictly reflects a stress‐adaptive program driven by inflammation and altered genome maintenance, rather than an underlying global enrichment of stem‐like transcriptional properties.

### 3.6. Phylogenetic Analysis and Evolutionary Conservation of ERCC1

**Figure 6:**
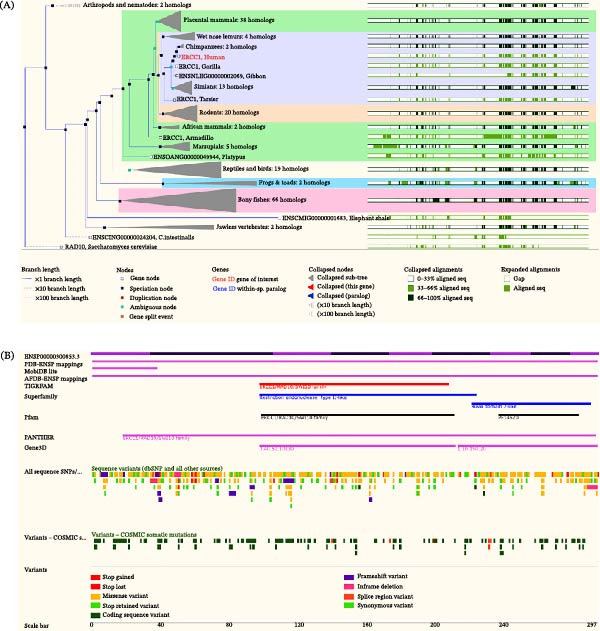
Phylogenetic and evolutionary conservation analysis of the *ERCC1* gene. (A) Phylogenetic tree (GeneTree) of the *ERCC1* gene family, illustrating evolutionary relationships ranging from arthropods and vertebrates to mammals; branch lengths represent evolutionary distances. (B) Multispecies protein sequence alignment highlighting the high conservation of key functional domains of *ERCC1*.

A GeneTree was constructed to trace the evolutionary history and sequence conservation of the ERCC1 gene across diverse taxa. The resulting phylogenetic tree illustrates the relationships between ERCC1 orthologs and paralogs, with branch lengths representing evolutionary divergence and nodes capturing speciation or duplication events, ranging from arthropods and nematodes to placental mammals (Figure 6A). A corresponding domain alignment underscores the strict conservation of critical structural units, particularly the core DNA‐binding and helix‐hairpin‐helix domains, validating ERCC1’s fundamental biological indispensability across the analyzed species (Figure 6B).

### 3.7. Structural Modeling and Interaction Analysis of ERCC1 Variants

While our transcriptomic data isolated the aberrant expression of ERCC1 as a key quantitative feature in endometriosis, functional impairment of DNA repair complexes frequently arises from qualitative structural defects dictated by genetic variation. Although the specific genotype status of the GSE6364 cohort is unavailable, we hypothesized that in individuals carrying high‐risk hypomorphic variants, structural instability would synergize with the observed transcriptional dysregulation to collapse repair capacity.

**Figure 7:**
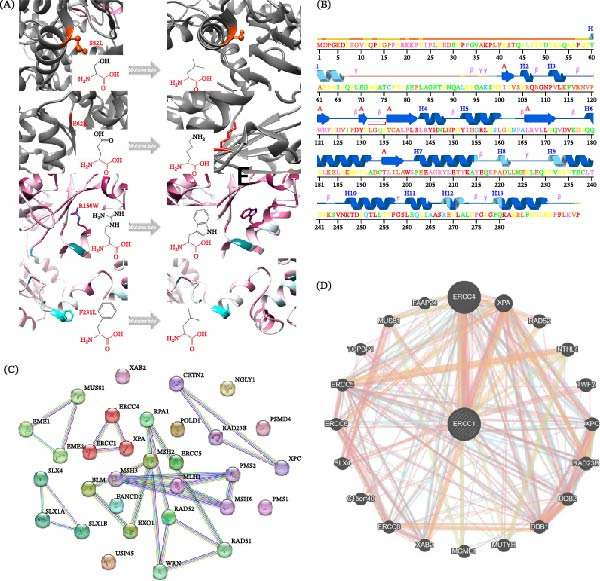
Structural modeling and interaction analysis of high‐risk ERCC1 SNPs. (A) Three‐dimensional structures of wild‐type and high‐risk SNP mutant (S52L and F231L) ERCC1 proteins predicted via homology modeling; detailed views demonstrate changes in side‐chain size and hydrophobicity caused by amino acid substitutions. (B) Secondary structure prediction of the ERCC1 protein sequence, showing the distribution of α‐helices and β‐sheets. (C, D) Specific PPI network of ERCC1, highlighting its interaction with key proteins of the nucleotide excision repair pathway.

To test this, in silico structural analysis was executed to project the physicochemical impact of high‐risk coding SNPs onto the ERCC1 protein. Homology models comparing wild‐type against mutant residues, specifically S52L and F231L, demonstrated significant local alterations in side‐chain bulk and regional hydrophobicity (Figure 7A). Secondary structure predictions mapped the precise distribution of alpha‐helices, beta‐sheets, and coiled domains, contextualizing these spatial shifts (Figure 7B). Furthermore, generated PPI networks positioned ERCC1 as the central node coordinating downstream NER executioners, including ERCC4 (XPF) and XPA (Figure 7C,D). Notably, the F231L substitution localizes to a highly conserved interface directly implicated in the obligate heterodimerization with ERCC4, providing a structural mechanism by which such variations could compromise genomic maintenance under inflammatory stress.

## 4. Discussion

Endometriosis remains a chronic, estrogen‐dependent disorder with a substantial burden of pain, infertility, and postoperative recurrence, underscoring the need to move beyond purely anatomical explanations and toward the molecular determinants of lesion establishment and persistence [1, 2, 6–8]. Using an integrative framework that combined differential transcriptomics, machine‐learning prioritization, immune deconvolution, and structural modeling, we identified four robust feature genes in the eutopic endometrium: ERCC1, SOX3, the p75 neurotrophic receptor (p75NTR; encoded by NGFR), and FPR1. Together, these signals support a model in which selective inflammatory programs interface with genome‐maintenance capacity and immune remodeling in the eutopic endometrium, thereby preconditioning refluxed cells to survive and adapt within the peritoneal environment.

A defining property of endometriosis is its capacity to persist within an inflammatory and pro‐oxidant microenvironment. Periodic bleeding, hemoglobin breakdown, and sustained immune activation generate ROS and related electrophiles that can damage nucleic acids, proteins, and lipids [15, 16]. Oxidative DNA lesions and DNA adducts are particularly relevant, as they create replicative stress and mutagenic substrates unless efficiently resolved by repair pathways [17]. In parallel, inflammatory signaling can reinforce oxidative stress and alter cellular survival thresholds, enabling damaged cells to evade clearance and promoting a tissue state that resembles chronic wound repair [18]. In this context, the enrichment of inflammatory‐response signatures in the eutopic endometrium, coupled with the prioritization of genes that map to immune and genome‐stability functions, suggests that disease susceptibility involves not only the “soil” of the peritoneal cavity but also an intrinsically stress‐adapted “seed” at the level of eutopic endometrial biology [5–8, 18].

A central implication of our analysis is the potential involvement of NER capacity in shaping endometrial stress tolerance. ERCC1 forms an obligate heterodimeric endonuclease with XPF, which is required for the incision step of NER and participates in additional repair contexts that preserve genome integrity [19, 20]. In our dataset, ERCC1 emerged as a top feature gene, and evolutionary analysis indicated strong conservation across species, consistent with stringent functional constraints. Importantly, our in silico modeling further suggested that putative missense substitutions could alter local physicochemical properties within ERCC1 and potentially compromise interaction surfaces relevant to ERCC1–XPF complex formation or stability. Structural studies have shown that ERCC1–XPF recruitment and positioning are tightly coupled to repair‐complex architecture, providing a mechanistic basis by which subtle perturbations could reduce repair efficiency under sustained oxidative stress [21]. Conceptually, a partial reduction in repair capacity may be sufficient to shift cell fate decisions under chronic inflammation from apoptosis toward survival with tolerable levels of DNA damage, thereby favoring persistence and outgrowth, a principle aligned with broader DDR paradigms in chronic disease and cancer biology [22].

Although endometriosis is histologically benign, its invasive behavior and epidemiologic association with selected malignancies suggest that long‐term survival under genotoxic stress could have consequences for clonal selection and tissue adaptation [1, 2, 11, 12, 23]. Our findings do not establish malignant progression; however, they nominate ERCC1‐linked repair capacity as a plausible axis through which inflammatory stress could be transduced into altered genome maintenance. This potentially facilitates persistence and, in some contexts, creates permissive conditions for the acquisition of cancer‐associated molecular features reported in endometriosis‐associated neoplasia [23]. This interpretation is consistent with the broader concept that chronic inflammation serves as a tumor‐promoting context by increasing DNA damage and imposing selection for stress‐resistant cellular states [18, 22].

Beyond genome maintenance, our immune deconvolution results indicate that the eutopic endometrium in endometriosis is characterized by selective, rather than global, changes in immune composition, alongside substantial interindividual heterogeneity. Such heterogeneity is consistent with the clinical diversity of endometriosis and with the likelihood that immune remodeling reflects both systemic factors and the local endometrial milieu [24–26]. Methodologically, deconvolution approaches provide an efficient estimate of immune fractions from bulk transcriptomes, but they also highlight the need for orthogonal validation in cell‐resolved datasets [27]. Notably, the prioritized feature genes showed distinct immune‐association patterns—including links to plasma cells, dendritic‐cell activation states, and neutrophils—supporting the notion that these transcripts are embedded within organized immune networks rather than isolated expression shifts. These patterns align with contemporary views that endometriosis involves immune dysfunction affecting surveillance, the clearance of refluxed tissue, and the balance between tolerance and inflammation [24, 25].

Two prioritized genes further suggest a convergence between innate immunity and neuroimmune signaling. FPR1 encodes a formyl peptide receptor that regulates leukocyte chemotaxis and inflammatory signaling in response to diverse endogenous and microbial ligands, positioning it as a potential amplifier or organizer of myeloid trafficking and activation [28]. Meanwhile, P75NTR is a multifunctional neurotrophic receptor with well‐established roles in cell survival, apoptosis, and signaling integration, including pathways that intersect with inflammatory cues [29]. The association of P75NTR expression with neutrophil abundance in our cohort is consistent with a disease context in which neurotrophic and inflammatory networks cooperate to shape cellular recruitment and persistence. This is clinically relevant because endometriosis is increasingly recognized as a disorder with neoangiogenic components that correlate with pain phenotypes, and increased nerve‐fiber density has been documented in the endometrium of affected individuals [26]. In this framework, altered P75NTR signaling may plausibly contribute to the coupling of inflammation, sensory innervation, and tissue remodeling, while FPR1 may reflect coordinated innate immune trafficking within the eutopic endometrial environment.

Collectively, our data support a unifying model: Inflammatory activation in the eutopic endometrium is accompanied by selective immune remodeling and is coupled to gene signatures encompassing genome‐maintenance machinery. Under this model, refluxed endometrial cells entering an oxidative peritoneal milieu may be more likely to survive if they originate from an endometrial state characterized by (i) heightened inflammatory signaling, (ii) altered interactions with innate and adaptive immune compartments, and (iii) perturbed DNA repair capacity that shifts stress responses toward persistence [15–19, 22, 24, 25]. This hypothesis yields testable predictions. For example, ERCC1 perturbation should sensitize endometrial cells to oxidative DNA damage and alter apoptosis thresholds, while the comodulation of inflammatory mediators should modify these effects in a nonadditive manner. Similarly, functional perturbation of FPR1 or P75NTR should influence immune cell recruitment, cytokine outputs, and potentially neoangiogenic readouts linked to pain.

Because endometriosis and ovarian endometrioma in particular have been discussed in the context of progenitor‐like programs and stemness‐associated phenotypes, we additionally examined a stemness‐related ssGSEA score in the same cohort. Prior studies have described the aberrant expression of stemness‐associated factors in endometriotic tissues, supporting the biological relevance of this question [30, 31]. In our dataset, however, the absence of a significant overall increase after phase adjustment suggests that the present four‐gene signature should not be interpreted as evidence of a global bulk‐tissue stemness shift. Instead, the data are more compatible with menstrual‐phase‐sensitive heterogeneity or with changes confined to a smaller subpopulation that cannot be resolved via bulk transcriptomics.

The existing literature provides differing levels of support for the four prioritized biomarkers. For ERCC1, previous evidence has mainly emerged from NER susceptibility studies rather than lesion‐focused expression analyses [14]. By contrast, NGFR/P75NTR has clearer lesion‐level support: NGFR/p75‐positive nerve fibers have been described in ovarian endometriomas, and p75 receptor expression has been documented in endometriotic lesions within neurotrophin‐related pain biology studies [32–34]. FPR1‐related signaling has likewise been implicated in endometriosis, including a surgically induced mouse model where the loss of Fpr1 attenuated lesion growth and pain behavior [35, 36]. Direct evidence for SOX3 in ovarian endometrioma remains limited, although it has been linked to invasion and stem cell–like behavior in endometrial cancer models [37]. Taken together, these reports suggest that NGFR and FPR1 are supported by existing endometriosis literature, ERCC1 is biologically anchored by broader DNA repair studies, and SOX3 may represent a comparatively novel candidate emerging from the present integrative analysis.

## 5. Conclusion

In conclusion, our integrative analyses nominate a compact, reproducible gene signature in the eutopic endometrium and support a model in which inflammatory activation is coupled to selective immune remodeling and altered genome‐maintenance capacity. In particular, ERCC1 emerges as a mechanistically plausible node linking chronic inflammatory stress to DNA repair competence, potentially lowering the barrier for cellular persistence in endometriosis. These findings provide a focused set of targets for experimental validation and lay a foundation for developing mechanism‐informed biomarkers and therapeutic strategies aimed at disrupting stress‐adaptive pathways that facilitate lesion establishment and recurrence. The exploratory stemness analysis further refines this interpretation by indicating that these signals are unlikely to reflect a uniform bulk‐tissue stemness enrichment.

## 6. Limitations

There are several limitations to this study. First, the candidate feature genes were prioritized using bioinformatics and machine learning approaches and have not yet been validated in independent clinical cohorts or experimental in vitro/in vivo models. Second, the use of bulk transcriptomic data limits the resolution of cell‐type‐specific expression programs and obscures spatial heterogeneity within endometriosis lesions. Third, the ERCC1 missense variants analyzed structurally were obtained from public variant databases rather than from paired germline sequencing of the transcriptomic cohort; direct genotype‐phenotype associations must be validated in future cohorts with matched sequencing and expression data. Fourth, because GSE6364 contains only eutopic endometrial biopsies, we were unable to compare the identified signals directly with native ovarian tissue or ovarian endometrioma lesions within the same analytical framework. Fifth, the added stemness‐related ssGSEA analysis was exploratory and performed on bulk expression data; therefore, cell‐type–specific progenitor‐like populations may still have been obscured and will require subsequent validation in single‐cell datasets.

## Author Contributions

Juan Wang formulated the research questions and drafted the manuscript for submission. Zhongmin Wang contributed to drafting the submitted manuscript. Wangshu Li assisted in data analysis and performed statistical analysis. Jiuxiang Feng designed and conducted the analysis.

## Funding

This work is supported by the Third Batch of Dean’s Funds for Dalian Women and Children’s Group (Grant 2024‐FEJY‐YZ‐12), the Dalian City Outstanding Young Science and Technology Talent Program (Grant 2023RY020), and the Liaoning Provincial Natural Science Foundation (Grant A2125) 2025.

## Conflicts of Interest

The authors declare no conflicts of interest.

## Data Availability

The data that support the findings of this study are openly available at https://www.ncbi.nlm.nih.gov.
